# Historical and contemporary range expansion of an invasive mussel, *Semimytlius algosus*, in Angola and Namibia despite data scarcity in an infrequently surveyed region

**DOI:** 10.1371/journal.pone.0239167

**Published:** 2020-09-11

**Authors:** Kevin C. K. Ma, Gerardo I. Zardi, Christopher D. McQuaid, Katy R. Nicastro

**Affiliations:** Department of Zoology and Entomology, Rhodes University, Grahamstown, Eastern Cape, South Africa; University of Sydney, AUSTRALIA

## Abstract

Understanding the spread of invasive species in many regions is difficult because surveys are rare. Here, historical records of the invasive marine mussel, *Semimytilus algosus*, on the shores of Angola and Namibia are synthesised to re-construct its invasive history. Since this mussel was first discovered in Namibia about 90 years ago, it has spread throughout the western coast of southern Africa. By the late 1960s, the species was well established across a range of 1005 km of coastline in southern Angola and northern Namibia. Although only coarse spatial resolution data are available since the 1990s, the distribution of *S*. *algosus* clearly increased substantially over the subsequent decades. Today, the species is distributed over 2785 km of coastline, appearing in southern Namibia in 2014, whence it spread across the border to northern South Africa in 2017, and in northern Angola in 2015. Conspicuously, its current range appears to be relatively contiguous across at least 810 km of shore in southern Angola and throughout Namibia, with isolated, spatially disjunct occurrences towards the southern and northern limits of its distribution. Despite there being few occurrence records that are unevenly distributed spatially and temporally, data for the distributional patterns of *S*. *algosus* in Angola and Namibia provide invaluable insights into how marine invasive species spread in developing regions that are infrequently monitored.

## Introduction

Native to the Pacific coast of South America, the bisexual mussel (so called because most individuals are simultaneously hermaphroditic), *Semimytilus algosus* (Gould 1850), has spread across the south Atlantic to the Atlantic coast of South America [1] and the western coast of southern Africa [2–5]. Although the early invasion history of *S*. *algosus* in Argentina and South Africa has been well-documented [1, 5, 6], less is known of the invasion of the Angolan and Namibian coasts by this species. In Namibia, Lamy [7] was the first to report *S*. *algosus* (described as *Modiola pseudocapensis*) from Walvis Bay during an expedition between 1928 and 1929 and Barnard [8] was the second (as *Semimytilus* sp.) from Cape Cross in 1957 [4, 9]. The first widespread intertidal rocky shore surveys in this region (i.e., Angola and Namibia) was made in the late 1960s—some 40 years after its initial discovery—by Kensley and Penrith [3, 9–11] and Penrith and Kensley [12, 13], which revealed relatively widespread distribution of *S*. *algosus* in northern Namibia and a couple of records in southern Angola.

The exact date of the arrival of *S*. *algosus* in northern Namibia and the circumstances surrounding its introduction (e.g., vectors) are not known, though its arrival may have considerably preceded its discovery in the late 1920s [7]. Bivalve aquaculture activity (e.g., the transport of oyster spat) and the transatlantic slave trade (e.g., wooden ships) have been linked to the introduction of marine alien species within Africa and around the world [14–16]. In this case, however, the timing of the appearance of *S*. *algosus* in Namibia before the development of aquaculture makes it probable that the fouling of ship hulls and the transport of planktonic larvae through ballast water crossing the Atlantic were probably the primary vectors and pathway [4]. The spread of *S*. *algosus* along the African coastline may involve another vector, namely, natural dispersal. Although the transport of oyster spat is a likely vector given the history of continuous transfer of spat between shellfish culture sites and known association with the introduction of many other marine organisms in the region [15], recent molecular evidence has identified long-distance larval transport via coastal currents as another possible vector for the introduction of *S*. *algosus* from Namibia to South Africa [6, 17].

At invaded sites, *S*. *algosus* and the native brown mussel *Perna perna* (Linnaeus, 1758) inhabit the same shore heights, from mid-shore to the lower shore of the intertidal rocks [3, 10]. Arriving in Namibia around 1989, another alien mussel, *Mytilus galloprovincialis* Lamarck, 1819, invaded many of these rocky shore habitats but was distributed higher in the intertidal zone [18–22]. Recruitment in Namibian populations of *S*. *algosus* occurs throughout the year with a peak during the austral summer [23, 24], which is consistent with seasonal recruitment patterns observed in its native range [25]. Moreover, Reaugh-Flower et al. [24] found that recruitment varied across sites (accounting for 29% of the variation) and seasons (9.6%) but not year, and that recruitment intensity was significantly correlated to adult densities in these Namibian populations. Elsewhere in Africa, *S*. *algosus* is a more recent invader, having been discovered in South Africa in 2009 [5]. In South Africa, *S*. *algosus* co-occurs with other mussel species (e.g., the native, *Aulacomya atra*, the native *Choromytilus meridionalis*, the non-native *Mytilus galloprovincialis*, and the native *P*. *perna*) with similar shore height habitat segregation to that observed in Namibia [20–22, 26]. In South Africa, both *S*. *algosus* and *M*. *galloprovincialis* have pronounced effects on the structure and maintenance of benthic communities, including competitive and facilitative interactions [26–28] with native mussel species [18, 29–32].

The paucity of occurrence records, coupled with the lack of resources dedicated to long-term, routine, large-scale monitoring of marine invasions in developing jurisdictions such as in Angola and Namibia, makes it challenging to understand the invasion history of marine alien species and the true extent of their distributions and ecological impacts [33–35]. In the case of *S*. *algosus*, we synthesised all available time- and geo-referenced occurrence records (i.e., presence and non-presence data) to re-construct its invasion history on the western coast of southern Africa and estimate its northerly and southerly rate of spread.

## Materials and methods

Records from: (1) the primary literature, (2) technical reports and theses, (3) an online database, the Ocean Biodiversity Information System (OBIS; https://obis.org/), and (4) our own field observations were tabulated to determine historical changes in the overall distribution of *S*. *algosus* spreading in Angola and Namibia (S1 Appendix) and northern South Africa (i.e., a single presence record extracted from Ma et al. [36]). In June 2020, we searched the online database, Google Scholar (https://scholar.google.com/), for literature containing relevant Angolan and Namibian records using the genus name “*Semimytilus*” and “Africa” as key search terms. From the resulting collection of literature (n = 265 articles), additional and obscure primary literature, technical reports, and student theses referenced therein were also examined for records.

Occurrence records were categorised as either ‘present’ or ‘not detected’. If there were no mentions of *S*. *algosus* from extensively surveyed sites, then the species was categorised as ‘not reported’. Years were all based on the date of collection or observation (DOC), except for a couple of records in which this date remained elusive as of this writing. For instance, the record from Luanda was based on the date of publication (DOP) of the 2015 study cited by Pestana et al. [37].

A total of 21 records of *S*. *algosus* from Namibia and none from Angola were found searching with OBIS. Eight of these records were from the Mollusc Collection at the South African Museum [38] and 13 from the West Coast Biodiversity Survey [39]. From this database, a record of *S*. *algosus* from Swakopmund (part of the Mollusc Collection dataset) was not associated with a year. Interestingly, this particular record from Swakopmund was also referred to in papers by Kensley and Penrith [3, 11] and Penrith and Kensley [13]; therefore, we backdated this record to as early as 1970 (i.e., the DOP of the earliest publication that cited this record).

In 2010 and 2011, observations of *S*. *algosus* in the field were made at four Namibian sites: Möwe Bay (19°22'23"S; 12°42'20"E), Swakopmund (22°40'27"S; 14°31'13"E), Walvis Bay (22°53'37"S; 14°26'18"E), and Lüderitz (26°37'56"S; 15°09'07"E). At these sites, *S*. *algosus* inhabited rocky substrata in the lower intertidal zone forming dense, mono-layered mussel beds at wave-exposed to moderately exposed sites. Multi-layered beds of other mussel species—namely *M*. *galloprovincialis* and *P*. *perna*—were also present at these sites. No permission was required to access these sites, no animals were physically removed from the habitat for this study, and no protected species were disturbed while making field observations.

## Results and discussion

### Overall distribution

**Fig 1:**
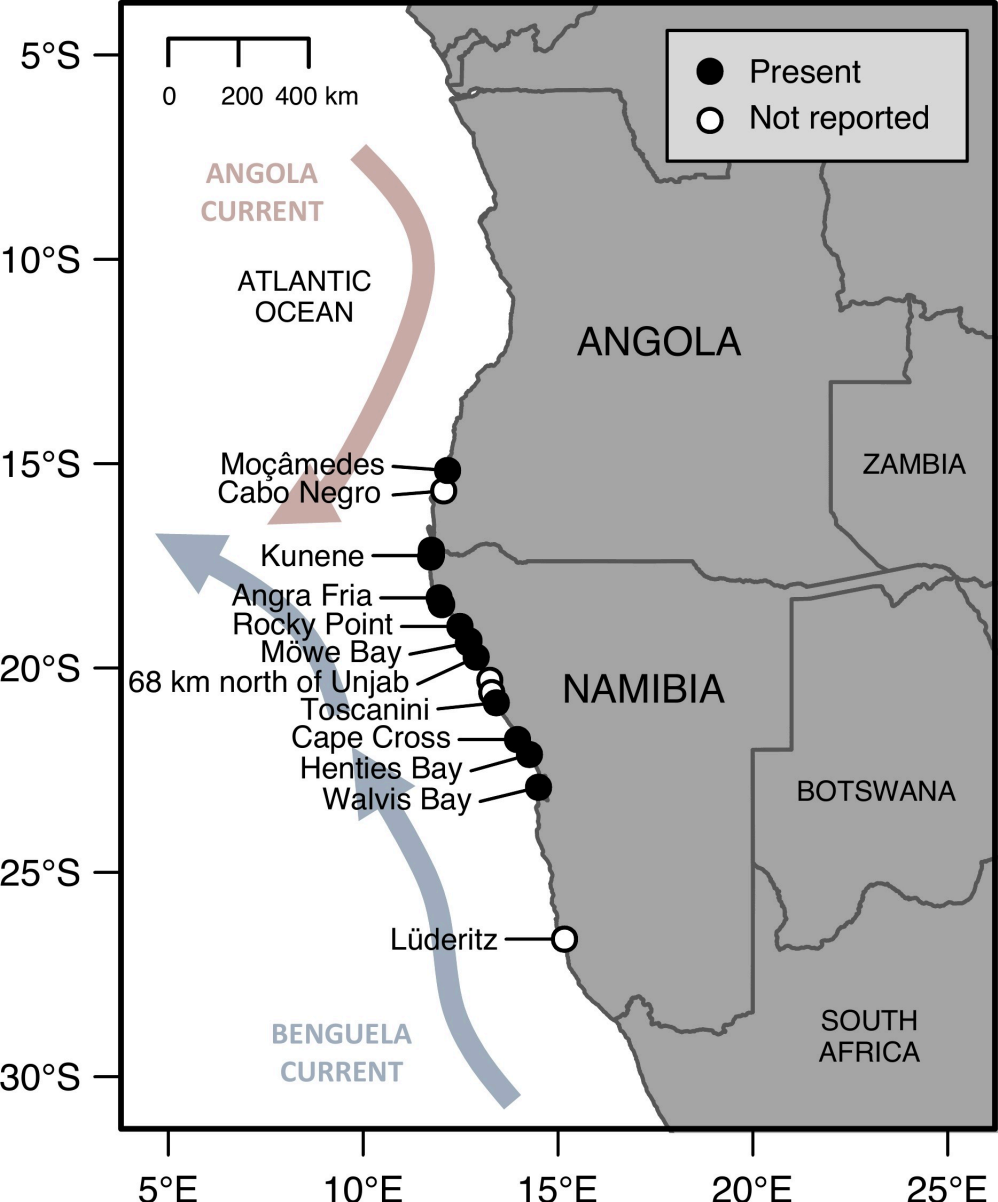
Distribution of *Semimytilus algosus in* Angola and Namibia by 1969. Broad direction of coastal currents, the Angola Current (pink arrow) and the Benguela Current (blue arrow) are indicated on the map. Data can be found in S1 Appendix.

**Fig 2:**
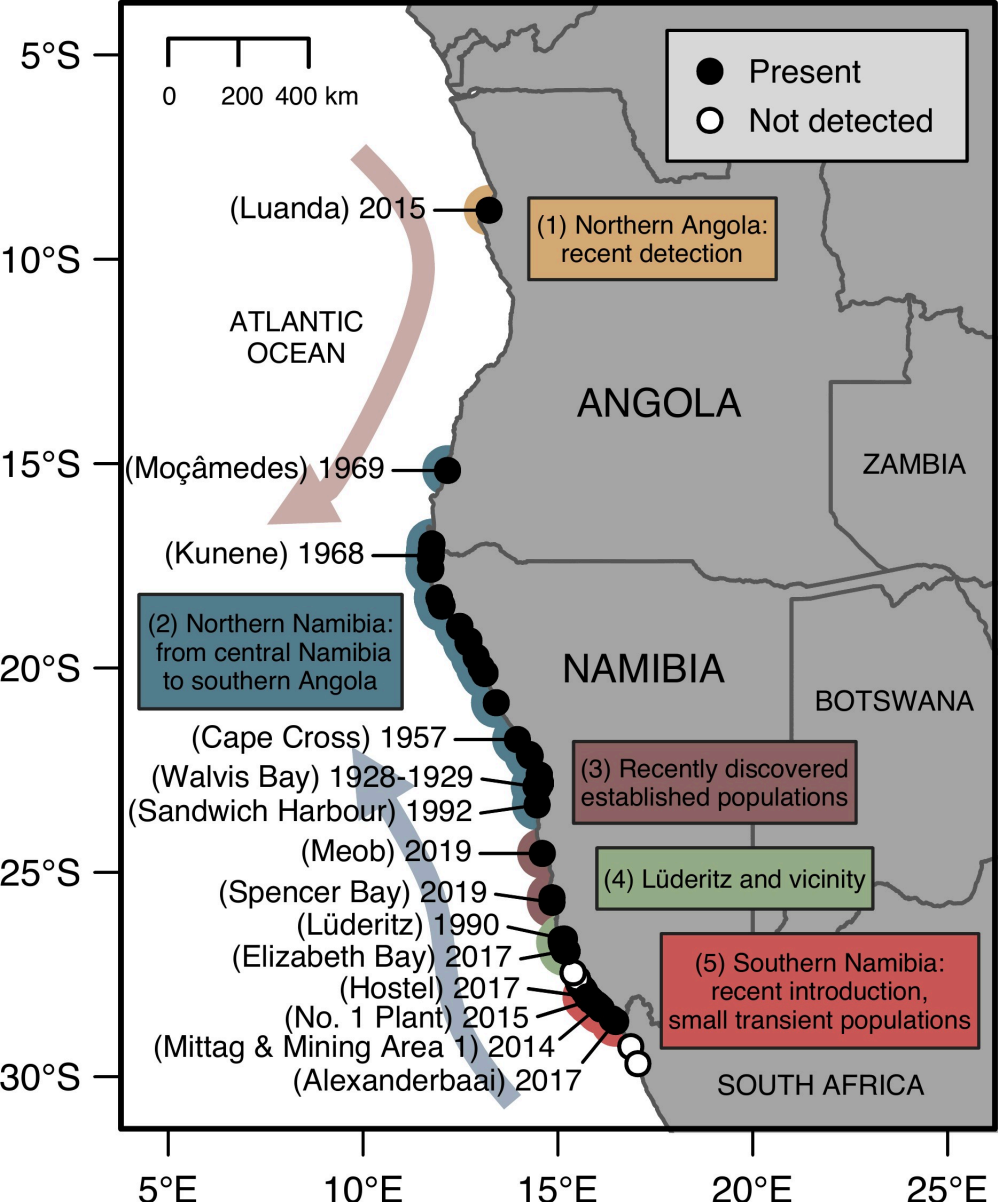
**Distribution of *Semimytilus algosus* in Angola and Namibia by 2019 showing (1) a recent detection in northern Angola, (2) historical spread in northern Namibia (ranging from central Namibia to southern Angola), (3) records from a previously unsurveyed region consisting of relatively established populations, (4) records from Lüderitz and vicinity consisting of small transient populations, and (5) a recent introduction to southern Namibia and local spread (e.g., spreading as far south as Alexanderbaai in northern South Africa).** Years mark temporal changes in distribution. Broad direction of coastal currents, the Angola Current (pink arrow) and the Benguela Current (blue arrow) are indicated on the map. Data from Angola and Namibia can be found in S1 Appendix; data from northern South Africa were extracted Ma et al. [36].

A total of 110 presence records and 255 non-presence (i.e., ‘not detected’ or ‘not reported’) records of *S*. *algosus* reported from Angola and Namibia and dated between 1928 and 2019 were compiled (S1 Appendix). The species was well established in northern Namibia by the late 1960s (Fig 1). From the 1990s to the present, the species appeared to be introduced to localised regions of the coast (Fig 2). Over the past half-century, the species’ range expanded from 730 (in 1968) to 2785 km (in 2019) along the west coast of Africa, ranging from Luanda in northern Angola [37] to Alexanderbaai in northern South Africa [36], which is 3 km south of the border between Namibia and South Africa (Table 1; Fig 2). Assuming no local extinctions, *S*. *algosus* occurrences are currently concentrated across a distance of 810 km of shore from Kunene (5 km north of the river mouth in Angola) to Sandwich Harbour (Namibia) with outlying (i.e., disjunct) occurrences at both the northern and southern limits of its coastal range (Fig 2). Based on the African occurrence records from this study and Ma et al. [36], *S*. *algosus* is presently distributed as an invasive species across 25°36' of latitude (from 8°48'S to 34°24'S; across 3885 km of coast). At this stage, this is substantially smaller than the native range of 36°16' of latitude (from 0°56'S to 37°12'S) on the Pacific coast of South America [1].

**Table 1:** Increase in the distributional range (spread) and the estimated rate of spread of *Semimytilus algosus* in Angola and Namibia.

**Year**	Observed spread (km)	Overall range (km)	Range spreading from Walvis Bay (km)	Range spreading in southern Namibia (km)	Rate of spread (km yr^-1^)	Cumulative rate of spread (km yr^-1^)	Remarks
1928–1929							First record from Walvis Bay
1957	—	165^a^	165^a^		—	—	Second record from Cape Cross
1968	—	730	730		—	—	Range from Kunene to Walvis Bay
1969	275 (N)	1005	1005		275 (N)	275 (N)	Range from Moçâmedes to Walvis Bay
1970							Records within known range
1978							Records within known range
1979							Records within known range
1990	—	1585	No change		—	—	New records from Lüderitz and Elizabeth Bay^b^
1992	80 (S)	No change	1085		3.3 (S)	3.3 (S)	Range from Moçâmedes to Sandwich Harbour
1995							Records within known range
1996							Records within known range
1997							Records within known range
1998							Records within known range
1999							Records within known range
2002							Records within known range
2004							No new records
2005							No new records
2006							No new records
2007							Records within known range^b^
2008							No new records
2009							No new records
2010							Records within known range
2011							Records within known range
2012							No new records
2013							No new records
2014	—	1775	No change	9^a^	—	—	New records from Mining Licence Area 1 and Mittag
2015	26 (N)	2735	No change	35	26 (N)	26 (N)	New records from Luanda and No. 1 Plant
2016							Records within known range
2017	12 (N), 50 (S)	2785	No change	97	6 (N), 16.7 (S)	12.7 (N), 16.7 (S)	New records from Hostel and Alexanderbaai^c^
2018							Records within known range
2019							New records from Meob and Spencer Bay^d^

N = northerly direction; S = southerly direction.

^a^ Value based on only two records

^b^ Along-shore range from Lüderitz to Elizabeth Bay was at least 50 km in 1990 and about 70 km in 2007

^c^ Alexanderbaai is in northern South Africa, about 3 km south of the border between Namibia and South Africa (see Ma et al. [36])

^d^ Records Meob and Spencer Bay were from a region that was not previously surveyed

### Invasion history: 1928 to 2019

Between 1928 and 1957, only two records of *S*. *algosus* were documented from Namibia: the first from Walvis Bay [4, 7] and the second from Cape Cross [8, 9]. The native brown mussel *P*. *perna* (described as *Mytilus perna* ranging from Morocco to the Cape of Good Hope) was the only mytilid species treated by Nickés [40] in a taxonomic monograph of molluscs ranging from Mauritania to Angola. The absence of *S*. *algosus* in this 1950 monograph suggests that the species had not yet invaded, or at least had not yet been detected in Angola.

In Angola and Namibia, the scale of *S*. *algosus* occurrence and distribution eventually came into focus after a series of surveys were done in the late 1960s [3, 9–13]. Initially, the species was reported to range from Rocky Point to Swakopmund, a distance of 485 km of coast [13]. Despite documenting this species at two localities (namely, north of Kunene and Moçâmedes) in southern Angola, its distribution was updated to range only from Angra Fria to Swakopmund, a distance of 583 km [9–11]. However, several years later, its distribution was eventually modified to reflect its northern occurrence in Moçâmedes with a reported coastal range of 975 km [3]. Deceptively, these along-shore distributional patterns do not reflect actual spread over this time period but how new information on its range was reported in the literature as sampling effort increased.

Upon closer examination of *S*. *algosus* occurrences in the late 1960s (Table 1), its distribution in 1969 was even more extensive than originally reported by Kensley and Penrith [9–11] and Penrith and Kensley [13], ranging across 730 km of coast from Kunene to Walvis Bay. In 1969, a single juvenile specimen of *S*. *algosus* was collected in Moçâmedes, which suggests that the species was a recent (i.e., contemporary to the 1960s) introduction to southern Angola. This expansion into Angola, thus, increased its range to 1005 km of shore from Moçâmedes to Walvis Bay (Table 1; Fig 1). This pattern of range expansion is a better reflection of the species’ spread in the region in the late 1960s, which is notable for (1) the rapid rate of spread and (2) the breaching of the biogeographic boundary from the Namib to the Angolan ecoregions [41–43]. Additionally, some intertidal sites were known to have been extensively surveyed in the late 1960s yet, conspicuously, no reports of *S*. *algosus* were documented from these sites [9, 10, 12], suggesting that its distribution was patchy across its range and that it was absent from the southern coast of Namibia (Fig 1).

Records of *S*. *algosus* from the 1970s—which included the Swakopmund record from the South African Museum’s Mollusc Collection—were all geo-referenced to sites within the then known range of the species [3, 11, 13, 38].

In 1990, *S*. *algosus* was detected in Lüderitz and Elizabeth Bay, which was about 530 km south of its previously known range [36, 44–46]. As of 1990, the 530 km of coastline separating the southern population in Lüderitz with the northern population in Walvis Bay had not been surveyed for *S*. *algosus*. The appearance of the species in Lüderitz and Elizabeth Bay could be either: (1) a gradual expansion of the range originating from Walvis Bay over many decades, or (2) a sudden introduction event. In the former scenario, the species would be expected to occur in the unsurveyed region. In the latter scenario, the date of introduction would occur sometime in the 1970s or the 1980s because the species was not reported from studies in Lüderitz and vicinity in the late 1960s [12]. Interestingly, the distributional gap between Walvis Bay and Lüderitz is also where the biogeographic transition between the Namib and Namaqua ecoregions is situated [41, 47].

Chronologically, the next occurrence in northern Namibia was recorded in 1992 [38], which expanded its distribution further south by a further 80 km (Table 1). Subsequently, records of *S*. *algosus* between 1995 and 2013—including our own field surveys—were all geo-referenced to sites within the known range of the species in northern Namibia and in the vicinity of Lüderitz [6, 17, 23, 24, 39, 48].

Elsewhere in Africa, *S*. *algosus* was documented in Mozambique and South Africa in 2008 and 2009, respectively. In Mozambique, the species was reported from Inhaca by the Kenya Marine and Fisheries Research Institute [49]. Given that this represented the only record of this species on the eastern coast of Africa, monitoring and independent confirmation of its occurrence in Mozambique are recommended. In South Africa, this species was discovered in Elandsbaai (i.e., ca. 835 km south of Lüderitz) and, by 2010, exhibited a South African range of 500 km of coast from Groenriviermond and Bloubergstrand [5].

Off-shore benthic surveys along the coast of Angola that were done in 2004 and, again, in 2011 resulted in the collection of four mytilid specimens [50, 51]. Unfortunately, none of these mussels were identified to species [50].

From 2004 to 2019, the region of southern Namibia was monitored annually for *S*. *algosus* across a total of 21 sites, although not all sites were surveyed each year [36, 52–57]. The monitored region extended from Wolf Bay, which is between Lüderitz and Elizabeth Bay, to Mittag, which is near the border between Namibia and South Africa. This corporate monitoring programme revealed that the species was no longer detected in the area around Lüderitz and Elizabeth Bay for 13 years until 2017. The apparent disappearance and re-appearance of the species from this area (i.e., Lüderitz and vicinity) suggests that (1) the local populations went extinct by 2004 and were re-introduced in 2017 or (2) the local population persisted at low numbers (or was restricted to the subtidal zone), evading detection by human observers until 2017. The former scenario is inconsistent with the report of the species from Lüderitz in 2007 [6, 17]. The latter scenario is more likely given the low abundance (< 0.5% cover) at intertidal sites where the species was observed, coupled with the transient nature of its occurrence at a given site [36].

In 2014, this corporate monitoring program detected populations in southern Namibia near the border between Namibia and South Africa [6, 53]. This southern population expanded its range northwards by 26 km (in 2015) and by another 12 km (in 2017), and southwards by 50 km (in 2017), resulting in a total along-shore range of 97 km in 2017 (Table 1). The absence of this species in areas immediately to the north and south of this area (i.e., the disjunct pattern of distribution; Fig 2), coupled with the bi-directional pattern of spread, suggests that the invasion of this coastal region arose from an introduction event rather than a gradual southerly spread from Lüderitz or northerly spread from South Africa.

In 2015, *S*. *algosus* was observed in Luanda, northern Angola [37], which is about 960 km north of Moçâmedes (Fig 2). This observation from Luanda is significant because no new Angolan observations north of Kunene had been made since 1969 (Table 1). To our knowledge, the coastal region between Luanda and Moçâmedes has not been monitored for the species. Despite the paucity of occurrence data from Angola, we speculate that this observation likely represents a relatively recent introduction event to northern Angola, associated with shipping activities given that the species was detected few kilometres from a port [37]. Luanda and vicinity should be monitored for local persistence or extinction to better track changes in *S*. *algosus* distribution in the region.

A large-scale survey of the Namibian coast mostly contributed new records within the known range of *S*. *algosus* and, importantly, in a previously unsurveyed region [20–22]. This previously unsurveyed region comprises a stretch of ca. 500 km of coast south of Sandwich Harbour near Walvis Bay and north of Lüderitz. The survey by Kreiner et al. [23] provided new records from Meob and Spencer Bay. The presence of relatively established populations at these sites can be explained by either (1) a gradual southerly expansion from Walvis Bay over many decades since 1928–1929, (2) a gradual northerly expansion from Lüderitz over many decades since 1990, or (3) a combination of both. Unfortunately, there is not enough information to speculate on the likely pattern(s) of colonisation for this coastal region linking Sandwich Harbour and Lüderitz.

### Concluding remarks

Despite low and uneven sampling effort across space and through time, resulting in a dearth of occurrence records in a developing region, here, we re-constructed the 90-year invasion history of *S*. *algosus* spreading in Angola and Namibia. Although we do not possess a perfect understanding of the invasion history of *S*. *algosus* along the western coast of southern Africa, this historical baseline information can provide guidance to the management of marine invasive species in this region [37]. For example, our data suggest that previously unoccupied areas have recently been colonised through *de novo* introductions rather the spread of existing populations, and that practitioners and stakeholders should focus their monitoring efforts on *S*. *algosus* near the northern and southern limits of its established distribution. Initially, the rate of spread of this species was relatively high (275 km yr^-1^) but, since the 1990s, spread remained one to two orders of magnitude lower (3.3–26 km yr^-1^; Table 1). Similarly, the invasive mussel, *Mytilus galloprovincialis* exhibited rapid spread (115 km yr^-1^) during the early stages of its invasion of the west coast of South Africa [18]. After the initial rapid range expansion, rates of *S*. *algosus* spread in Angola and Namibia were comparable to the estimated rates (7.5–40 km yr^-1^) of *S*. *algosus* spread in South Africa [36].

Increased effort in monitoring for marine invasions can decrease the probability of false negative detections, especially if invasive species are present at very low abundances. How much effort is required to detect real changes in distributional patterns confidently (e.g., disappearance and re-appearance of species in particular regions) for rare invasive species is still difficult to know. Yet, standardised sampling protocol over a large spatial coverage, frequent sampling over a long time period (e.g., annual monitoring), or a combination of both can help overcome false negative detections and greatly improve our understanding of spatio-temporal invasion patterns [58, 59]. Further compounding the problem, monitoring for marine biological invasions can be particularly challenging in developing regions where resources are limited as this can affect the quality and availability of data [33–35].

An accurate historical and contemporary understanding of the distribution, spread, and drivers of marine invasions can enhance our ability to protect ecologically and commercially important resources, control the spread of invasive species, and model the processes regulating invasions [19, 60]. Ecologically, the spread of marine alien species, such as *S*. *algosus* in Angola and Namibia, can serve as a proxy indicator of abiotic and biotic changes in marine systems because the distribution of spreading species (both alien and native species) may be strongly linked to changing environmental conditions [61, 62]. The arrival and spread of invasive species have dramatic effects on the recipient communities and can be particularly marked in the case of ecosystem engineers such as marine mussels [18, 31]. In particular, the invasion of southern Africa by marine mussels (*S*. *algosus*, *M*. *galloprovincialis*) has had ecological consequences ranging from replacement of indigenous species as the dominant mussel to effects on infauna associated with mussel beds and increases in the abundance of marine birds that feed on mussels [18, 29–32, 63]. Although it grows relatively slowly and exhibits high mortality rates that are compensated by high recruitment success, *S*. *algosus* supports fewer limpet species compared to other sympatric mussel species due to its smaller shell size and, in turn, reduced grazing allows algae to proliferate [64]. Nevertheless, interactions between alien and native ecosystem engineers (e.g., mytilid mussels) may often result in substantial changes to the structure and composition of the species assemblage [65–67].

## Supporting information

S1 Appendix Occurrence records of *Semimytilus algosus* in Angola and Namibia. Present = observation or collection was made; not reported = relatively detailed observations of mussel species other than *S*. *algosus* were reported from a surveyed site; not detected = no observations or collections were made.
(DOCX)
