# Supplementary material for: Historical and contemporary range expansion of an invasive mussel, Semimytlius algosus, in Angola and Namibia despite data scarcity in an infrequently surveyed region
Source: PLoS One. 2020 Sep 11;15(9):e0239167. doi: 10.1371/journal.pone.0239167 (PMC7485899; doi:10.1371/journal.pone.0239167)
Supplement: S1 Appendix — Present = observation or collection was made; not reported = relatively detailed observations of mussel species other than S. algosus were reported from a surveyed site; not detected = no observations or collections were made. (DOCX) [file pone.0239167.s001.docx]

**S1 Appendix.** **Occurrence records of *Semimytilus algosus* in Angola and Namibia.** Present = observation or collection was made; not reported = relatively detailed observations of mussel species other than *S. algosus* were reported from a surveyed site; not detected = no observations or collections were made.

| Year | Site | Latitude | Longitude | Occurrence | Source |
| --- | --- | --- | --- | --- | --- |
|  |  |  |  |  |  |
| 1928–1929 | Walvis Bay | 22°55'S | 14°30'E | Present | [7], [4] |
|  |  |  |  |  |  |
| 1957 | Cape Cross | 21°45'S | 13°37'E | Present | [8], [9] |
|  |  |  |  |  |  |
| 1968 | 6–8 km south of Kunene | 17°15'S | 11°45'E | Not reported | [9] |
|  | 5 km south of Kunene | 17°17'S | 11°44'E | Present | [3] |
|  | Angra Fria | 18°17'S | 11°57'E | Present | [9] |
|  | Cape Frio | 18°26'S | 12°00'E | Present | [9] |
|  | Cape Frio | 18°27'S | 12°01'E | Present | [38] |
|  | Rocky Point | 18°59'S | 12°29'E | Present | [9], [13] |
|  | 68 km north of Unjab | 19°44'S | 12°54'E | Present | [9] |
|  | Honolulu | 20°36'S | 13°18'E | Not reported | [9] |
|  | Toscanini | 20°51'S | 13°25'E | Present | [9] |
|  | Lüderitz and vicinity | 26°38'S | 15°10'E | Not reported | [12] |
|  |  |  |  |  |  |
| 1969 | Moçâmedes | 15°10'S | 12°10'E | Present | [10], [11] |
|  | Cabo Negro | 15°40'S | 12°04'E | Not reported | [10] |
|  | 15 km north of Kunene | 17°07'S | 11°45'E | Present | [10] |
|  | Kunene | 17°15'S | 11°45'E | Present | [38] |
|  | 5 km south of Kunene | 17°17'S | 11°44'E | Present | [3] |
|  | Rocky Point | 18°59'S | 12°29'E | Present | [9], [13] |
|  | Möwe Bay | 19°20'S | 12°43'E | Present | [9] |
|  | Möwe Bay | 19°23'S | 12°42'E | Present | [38] |
|  | Torra Bay | 20°18'S | 13°15'E | Not reported | [9] |
|  | Henties Bay | 22°07'S | 14°16'E | Present | [38] |
|  | Lüderitz and vicinity | 26°38'S | 15°10'E | Not reported | [12] |
|  |  |  |  |  |  |
| 1970 | Swakopmund | 22°42'S | 14°31'E | Present | [3], [38], [13]^a^, [11] |
|  |  |  |  |  |  |
| 1978 | 30 km north of Kunene | 16°58'S | 11°46'E | Present | [3]^b^ |
|  | 5 km south of Kunene | 17°17'S | 11°44'E | Present | [3]^b^ |
|  | Angra Fria | 18°17'S | 11°57'E | Present | [3]^b^ |
|  | False Cape Fria | 18°29'S | 12°01'E | Present | [3]^b^ |
|  |  |  |  |  |  |
| 1979 | 30 km north of Kunene | 16°58'S | 11°46'E | Present | [3]^b^ |
|  | 5 km south of Kunene | 17°17'S | 11°44'E | Present | [3]^b^ |
|  | Angra Fria | 18°17'S | 11°57'E | Present | [3]^b^ |
|  | False Cape Fria | 18°29'S | 12°01'E | Present | [3]^b^ |
|  |  |  |  |  |  |
| 1990 | Lüderitz | 26°42'30"S | 15°05'10"E | Present | [44]^c^ |
|  | Elizabeth Bay | 27°00'S | 15°14'E | Present | [46], [47] |
|  |  |  |  |  |  |
| 1992 | Sandwich Harbour | 23°21'S | 14°28'E | Present | [38] |
|  |  |  |  |  |  |
| 1995 | Mile Four, Swakopmund | 22°37'36"S | 14°31'28"E | Present | [23], [48], [24] |
|  | Badewanne, Swakopmund | 22°42'02"S | 14°31'31"E | Present | [23], [48], [24] |
|  | Langstrand North, Swakopmund | 22°49'11"S | 14°32'59"E | Present | [23], [48], [24] |
|  | Langstrand South, Swakopmund | 22°49'11"S | 14°32'59"E | Present | [48], [24] |
|  |  |  |  |  |  |
| 1996 | Mile Four, Swakopmund | 22°37'36"S | 14°31'28"E | Present | [23], [48], [24] |
|  | Badewanne, Swakopmund | 22°42'02"S | 14°31'31"E | Present | [23], [48], [24] |
|  | Langstrand North, Swakopmund | 22°49'11"S | 14°32'59"E | Present | [23], [48], [24] |
|  | Langstrand South, Swakopmund | 22°49'11"S | 14°32'59"E | Present | [48], [24] |
|  |  |  |  |  |  |
| 1997 | Mile Four, Swakopmund | 22°37'36"S | 14°31'28"E | Present | [48], [24] |
|  | Badewanne, Swakopmund | 22°42'02"S | 14°31'31"E | Present | [48], [24] |
|  | Langstrand North, Swakopmund | 22°49'11"S | 14°32'59"E | Present | [15], [24] |
|  | Langstrand South, Swakopmund | 22°49'11"S | 14°32'59"E | Present | [15], [24] |
|  |  |  |  |  |  |
| 1998 | Mile Four, Swakopmund | 22°37'36"S | 14°31'28"E | Present | [15], [24] |
|  | Badewanne, Swakopmund | 22°42'02"S | 14°31'31"E | Present | [15], [24] |
|  | Langstrand North, Swakopmund | 22°49'11"S | 14°32'59"E | Present | [15], [24] |
|  | Langstrand South, Swakopmund | 22°49'11"S | 14°32'59"E | Present | [15], [24] |
|  |  |  |  |  |  |
| 1999 | Mile Four, Swakopmund | 22°37'36"S | 14°31'28"E | Present | [15], [24] |
|  | Badewanne, Swakopmund | 22°42'02"S | 14°31'31"E | Present | [15], [24] |
|  | Langstrand North, Swakopmund | 22°49'11"S | 14°32'59"E | Present | [15], [24] |
|  | Langstrand South, Swakopmund | 22°49'11"S | 14°32'59"E | Present | [15], [24] |
|  |  |  |  |  |  |
| 2002 | 35 km south of Kunene | 17°34'12"S | 11°43'48"E | Present | [39] |
|  | Angra Fria | 18°17'24"S | 11°57'36"E | Present | [39] |
|  | False Cape Fria | 18°28'12"S | 12°01'12"E | Present | [39] |
|  | Rocky Point | 18°59'24"S | 12°28'48"E | Present | [39] |
|  | Möwe Bay | 19°21'36"S | 12°41'24"E | Present | [39] |
|  | Terrace Bay | 19°59'24"S | 13°01'48"E | Present | [39] |
|  | Students Bay | 20°07'48"S | 13°07'48"E | Present | [39] |
|  | Swakopmund | 22°40'12"S | 14°31'12"E | Present | [39] |
|  |  |  |  |  |  |
| 2004 | Wolf Bay | 26°48'27"S | 15°07'27"E | Not detected | [36]^e^ |
|  | Atlas Bay | 26°49'45"S | 15°08'21"E | Not detected | [36]^e^ |
|  | East Whale | 26°54'28"S | 15°09'47"E | Not detected | [36]^e^ |
|  | Elizabeth Bay Point, exposed | 26°55'27"S | 15°11'31"E | Not detected | [36]^e^ |
|  | Elizabeth Bay Point, semi-exposed | 26°55'21"S | 15°11'32"E | Not detected | [36]^e^ |
|  | South Jetty | 26°55'13"S | 15°11'31"E | Not detected | [36]^e^ |
|  | North Jetty | 26°55'06"S | 15°11'30"E | Not detected | [36]^e^ |
|  | Elizabeth Bay South | 26°56'08"S | 15°13'48"E | Not detected | [36]^e^ |
|  | Bogenfels | 27°27'26"S | 15°23'27"E | Not detected | [36]^e^ |
|  | Green Precipice | 27°47'22"S | 15°34'59"E | Not detected | [36]^e^ |
|  | Cabin | 27°36'26"S | 15°29'48"E | Not detected | [36]^e^ |
|  | Dernberg Bay | 27°42'37"S | 15°31'52"E | Not detected | [36]^e^ |
|  | Site 1 | 27°51'19"S | 15°38'52"E | Not detected | [36]^e^ |
|  | Panther Reef | 27°54'55"S | 15°41'05"E | Not detected | [36]^e^ |
|  |  |  |  |  |  |
| 2005 | Wolf Bay | 26°48'27"S | 15°07'27"E | Not detected | [36]^e^ |
|  | Atlas Bay | 26°49'45"S | 15°08'21"E | Not detected | [36]^e^ |
|  | East Whale | 26°54'28"S | 15°09'47"E | Not detected | [36]^e^ |
|  | Elizabeth Bay Point, exposed | 26°55'27"S | 15°11'31"E | Not detected | [36]^e^ |
|  | Elizabeth Bay Point, semi-exposed | 26°55'21"S | 15°11'32"E | Not detected | [36]^e^ |
|  | South Jetty | 26°55'13"S | 15°11'31"E | Not detected | [36]^e^ |
|  | North Jetty | 26°55'06"S | 15°11'30"E | Not detected | [36]^e^ |
|  | Elizabeth Bay South | 26°56'08"S | 15°13'48"E | Not detected | [36]^e^ |
|  | Bogenfels | 27°27'26"S | 15°23'27"E | Not detected | [36]^e^ |
|  | Green Precipice | 27°47'22"S | 15°34'59"E | Not detected | [36]^e^ |
|  | Cabin | 27°36'26"S | 15°29'48"E | Not detected | [36]^e^ |
|  | Dernberg Bay | 27°42'37"S | 15°31'52"E | Not detected | [36]^e^ |
|  | Site 1 | 27°51'19"S | 15°38'52"E | Not detected | [36]^e^ |
|  | Panther Reef | 27°54'55"S | 15°41'05"E | Not detected | [36]^e^ |
|  |  |  |  |  |  |
| 2006 | Wolf Bay | 26°48'27"S | 15°07'27"E | Not detected | [36]^e^ |
|  | Atlas Bay | 26°49'45"S | 15°08'21"E | Not detected | [36]^e^ |
|  | East Whale | 26°54'28"S | 15°09'47"E | Not detected | [36]^e^ |
|  | Elizabeth Bay Point, exposed | 26°55'27"S | 15°11'31"E | Not detected | [36]^e^ |
|  | Elizabeth Bay Point, semi-exposed | 26°55'21"S | 15°11'32"E | Not detected | [36]^e^ |
|  | South Jetty | 26°55'13"S | 15°11'31"E | Not detected | [36]^e^ |
|  | North Jetty | 26°55'06"S | 15°11'30"E | Not detected | [36]^e^ |
|  | Elizabeth Bay South | 26°56'08"S | 15°13'48"E | Not detected | [36]^e^ |
|  | Bogenfels | 27°27'26"S | 15°23'27"E | Not detected | [36]^e^ |
|  | Green Precipice | 27°47'22"S | 15°34'59"E | Not detected | [36]^e^ |
|  | Cabin | 27°36'26"S | 15°29'48"E | Not detected | [36]^e^ |
|  | Dernberg Bay | 27°42'37"S | 15°31'52"E | Not detected | [36]^e^ |
|  | Site 1 | 27°51'19"S | 15°38'52"E | Not detected | [36]^e^ |
|  | Panther Reef | 27°54'55"S | 15°41'05"E | Not detected | [36]^e^ |
|  |  |  |  |  |  |
| 2007 | Lüderitz | 26°38'S | 15°10'E | Present | [6]^d^, [17]^d^ |
|  | Wolf Bay | 26°48'27"S | 15°07'27"E | Not detected | [36]^e^ |
|  | Atlas Bay | 26°49'45"S | 15°08'21"E | Not detected | [36]^e^ |
|  | West Whale | 26°54'32"S | 15°09'39"E | Not detected | [36]^e^ |
|  | East Whale | 26°54'28"S | 15°09'47"E | Not detected | [36]^e^ |
|  | Elizabeth Bay Point, exposed | 26°55'27"S | 15°11'31"E | Not detected | [36]^e^ |
|  | Elizabeth Bay Point, semi-exposed | 26°55'21"S | 15°11'32"E | Not detected | [36]^e^ |
|  | South Jetty | 26°55'13"S | 15°11'31"E | Not detected | [36]^e^ |
|  | North Jetty | 26°55'06"S | 15°11'30"E | Not detected | [36]^e^ |
|  | Elizabeth Bay South | 26°56'08"S | 15°13'48"E | Not detected | [36]^e^ |
|  | Bogenfels | 27°27'26"S | 15°23'27"E | Not detected | [36]^e^ |
|  | Green Precipice | 27°47'22"S | 15°34'59"E | Not detected | [36]^e^ |
|  | Cabin | 27°36'26"S | 15°29'48"E | Not detected | [36]^e^ |
|  | Dernberg Bay | 27°42'37"S | 15°31'52"E | Not detected | [36]^e^ |
|  | Site 1 | 27°51'19"S | 15°38'52"E | Not detected | [36]^e^ |
|  | Panther Reef | 27°54'55"S | 15°41'05"E | Not detected | [36]^e^ |
|  | Hostel (H155-160) | 28°02'50"S | 15°45'56"E | Not detected | [52] |
|  | No. 1 Plant (H35-50) | 28°07'38"S | 15°51'00"E | Not detected | [52] |
|  | Kerbe Huk (K65-69) | 28°13'53"S | 15°59'01"E | Not detected | [52] |
|  | No. 2 Plant (M170-175) | 28°16'46"S | 16°02'45"E | Not detected | [52] |
|  | Mittag (M80-85) | 28°20'20"S | 16°06'24"E | Not detected | [52] |
|  |  |  |  |  |  |
| 2008 | Wolf Bay | 26°48'27"S | 15°07'27"E | Not detected | [36]^e^ |
|  | Atlas Bay | 26°49'45"S | 15°08'21"E | Not detected | [36]^e^ |
|  | West Whale | 26°54'32"S | 15°09'39"E | Not detected | [36]^e^ |
|  | East Whale | 26°54'28"S | 15°09'47"E | Not detected | [36]^e^ |
|  | Elizabeth Bay Point, exposed | 26°55'27"S | 15°11'31"E | Not detected | [36]^e^ |
|  | Elizabeth Bay Point, semi-exposed | 26°55'21"S | 15°11'32"E | Not detected | [36]^e^ |
|  | South Jetty | 26°55'13"S | 15°11'31"E | Not detected | [36]^e^ |
|  | North Jetty | 26°55'06"S | 15°11'30"E | Not detected | [36]^e^ |
|  | Elizabeth Bay South | 26°56'08"S | 15°13'48"E | Not detected | [36]^e^ |
|  | Bogenfels | 27°27'26"S | 15°23'27"E | Not detected | [36]^e^ |
|  | Green Precipice | 27°47'22"S | 15°34'59"E | Not detected | [36]^e^ |
|  | Cabin | 27°36'26"S | 15°29'48"E | Not detected | [36]^e^ |
|  | Dernberg Bay | 27°42'37"S | 15°31'52"E | Not detected | [36]^e^ |
|  | Site 1 | 27°51'19"S | 15°38'52"E | Not detected | [36]^e^ |
|  | Panther Reef | 27°54'55"S | 15°41'05"E | Not detected | [36]^e^ |
|  | Hostel (H155-160) | 28°02'50"S | 15°45'56"E | Not detected | [52] |
|  | No. 1 Plant (H35-50) | 28°07'38"S | 15°51'00"E | Not detected | [52] |
|  | Kerbe Huk (K65-69) | 28°13'53"S | 15°59'01"E | Not detected | [52] |
|  | No. 2 Plant (M170-175) | 28°16'46"S | 16°02'45"E | Not detected | [52] |
|  | Mittag (M80-85) | 28°20'20"S | 16°06'24"E | Not detected | [52] |
|  |  |  |  |  |  |
| 2009 | Wolf Bay | 26°48'27"S | 15°07'27"E | Not detected | [36]^e^ |
|  | Atlas Bay | 26°49'45"S | 15°08'21"E | Not detected | [36]^e^ |
|  | West Whale | 26°54'32"S | 15°09'39"E | Not detected | [36]^e^ |
|  | East Whale | 26°54'28"S | 15°09'47"E | Not detected | [36]^e^ |
|  | Elizabeth Bay Point, exposed | 26°55'27"S | 15°11'31"E | Not detected | [36]^e^ |
|  | Elizabeth Bay Point, semi-exposed | 26°55'21"S | 15°11'32"E | Not detected | [36]^e^ |
|  | South Jetty | 26°55'13"S | 15°11'31"E | Not detected | [36]^e^ |
|  | North Jetty | 26°55'06"S | 15°11'30"E | Not detected | [36]^e^ |
|  | Elizabeth Bay South | 26°56'08"S | 15°13'48"E | Not detected | [36]^e^ |
|  | Bogenfels | 27°27'26"S | 15°23'27"E | Not detected | [36]^e^ |
|  | Green Precipice | 27°47'22"S | 15°34'59"E | Not detected | [36]^e^ |
|  | Cabin | 27°36'26"S | 15°29'48"E | Not detected | [36]^e^ |
|  | Dernberg Bay | 27°42'37"S | 15°31'52"E | Not detected | [36]^e^ |
|  | Site 1 | 27°51'19"S | 15°38'52"E | Not detected | [36]^e^ |
|  | Panther Reef | 27°54'55"S | 15°41'05"E | Not detected | [36]^e^ |
|  | Hostel (H155-160) | 28°02'50"S | 15°45'56"E | Not detected | [52] |
|  | No. 1 Plant (H35-50) | 28°07'38"S | 15°51'00"E | Not detected | [52] |
|  | Kerbe Huk (K65-69) | 28°13'53"S | 15°59'01"E | Not detected | [52] |
|  | No. 2 Plant (M170-175) | 28°16'46"S | 16°02'45"E | Not detected | [52] |
|  | Mittag (M80-85) | 28°20'20"S | 16°06'24"E | Not detected | [52] |
|  |  |  |  |  |  |
| 2010 | Möwe Bay | 19°22'23"S | 12°42'20"E | Present | Present study |
|  | Swakopmund | 22°40'27"S | 14°31'13"E | Present | Present study |
|  | Walvis Bay | 22°53'37"S | 14°26'18"E | Present | Present study |
|  | Lüderitz | 26°37'56"S | 15°09'07"E | Present | Present study |
|  | Wolf Bay | 26°48'27"S | 15°07'27"E | Not detected | [36]^e^ |
|  | Atlas Bay | 26°49'45"S | 15°08'21"E | Not detected | [36]^e^ |
|  | West Whale | 26°54'32"S | 15°09'39"E | Not detected | [36]^e^ |
|  | East Whale | 26°54'28"S | 15°09'47"E | Not detected | [36]^e^ |
|  | Elizabeth Bay Point, exposed | 26°55'27"S | 15°11'31"E | Not detected | [36]^e^ |
|  | Elizabeth Bay Point, semi-exposed | 26°55'21"S | 15°11'32"E | Not detected | [36]^e^ |
|  | South Jetty | 26°55'13"S | 15°11'31"E | Not detected | [36]^e^ |
|  | North Jetty | 26°55'06"S | 15°11'30"E | Not detected | [36]^e^ |
|  | Elizabeth Bay South | 26°56'08"S | 15°13'48"E | Not detected | [36]^e^ |
|  | Bogenfels | 27°27'26"S | 15°23'27"E | Not detected | [36]^e^ |
|  | Cabin | 27°36'26"S | 15°29'48"E | Not detected | [36]^e^ |
|  | Dernberg Bay | 27°42'37"S | 15°31'52"E | Not detected | [36]^e^ |
|  | Site 1 | 27°51'19"S | 15°38'52"E | Not detected | [36]^e^ |
|  | Panther Reef | 27°54'55"S | 15°41'05"E | Not detected | [36]^e^ |
|  | Hostel (H155-160) | 28°02'50"S | 15°45'56"E | Not detected | [52] |
|  | No. 1 Plant (H35-50) | 28°07'38"S | 15°51'00"E | Not detected | [52] |
|  | Kerbe Huk (K65-69) | 28°13'53"S | 15°59'01"E | Not detected | [52] |
|  | No. 2 Plant (M170-175) | 28°16'46"S | 16°02'45"E | Not detected | [52] |
|  | Mittag (M80-85) | 28°20'20"S | 16°06'24"E | Not detected | [52] |
|  |  |  |  |  |  |
| 2011 | Möwe Bay | 19°22'23"S | 12°42'20"E | Present | Present study |
|  | Swakopmund | 22°40'27"S | 14°31'13"E | Present | Present study |
|  | Walvis Bay | 22°53'37"S | 14°26'18"E | Present | Present study |
|  | Lüderitz | 26°37'56"S | 15°09'07"E | Present | Present study |
|  | Wolf Bay | 26°48'27"S | 15°07'27"E | Not detected | [36]^e^ |
|  | Atlas Bay | 26°49'45"S | 15°08'21"E | Not detected | [36]^e^ |
|  | West Whale | 26°54'32"S | 15°09'39"E | Not detected | [36]^e^ |
|  | East Whale | 26°54'28"S | 15°09'47"E | Not detected | [36]^e^ |
|  | Elizabeth Bay Point, exposed | 26°55'27"S | 15°11'31"E | Not detected | [36]^e^ |
|  | Elizabeth Bay Point, semi-exposed | 26°55'21"S | 15°11'32"E | Not detected | [36]^e^ |
|  | South Jetty | 26°55'13"S | 15°11'31"E | Not detected | [36]^e^ |
|  | North Jetty | 26°55'06"S | 15°11'30"E | Not detected | [36]^e^ |
|  | Elizabeth Bay South | 26°56'08"S | 15°13'48"E | Not detected | [36]^e^ |
|  | Bogenfels | 27°27'26"S | 15°23'27"E | Not detected | [36]^e^ |
|  | Cabin | 27°36'26"S | 15°29'48"E | Not detected | [36]^e^ |
|  | Dernberg Bay | 27°42'37"S | 15°31'52"E | Not detected | [36]^e^ |
|  | Site 1 | 27°51'19"S | 15°38'52"E | Not detected | [36]^e^ |
|  | Panther Reef | 27°54'55"S | 15°41'05"E | Not detected | [36]^e^ |
|  | Hostel (H155-160) | 28°02'50"S | 15°45'56"E | Not detected | [52] |
|  | No. 1 Plant (H35-50) | 28°07'38"S | 15°51'00"E | Not detected | [52] |
|  | Kerbe Huk (K65-69) | 28°13'53"S | 15°59'01"E | Not detected | [52] |
|  | No. 2 Plant (M170-175) | 28°16'46"S | 16°02'45"E | Not detected | [52] |
|  | Mittag (M80-85) | 28°20'20"S | 16°06'24"E | Not detected | [52] |
|  |  |  |  |  |  |
| 2012 | Wolf Bay | 26°48'27"S | 15°07'27"E | Not detected | [36]^e^ |
|  | Atlas Bay | 26°49'45"S | 15°08'21"E | Not detected | [36]^e^ |
|  | West Whale | 26°54'32"S | 15°09'39"E | Not detected | [36]^e^ |
|  | East Whale | 26°54'28"S | 15°09'47"E | Not detected | [36]^e^ |
|  | Elizabeth Bay Point, exposed | 26°55'27"S | 15°11'31"E | Not detected | [36]^e^ |
|  | Elizabeth Bay Point, semi-exposed | 26°55'21"S | 15°11'32"E | Not detected | [36]^e^ |
|  | South Jetty | 26°55'13"S | 15°11'31"E | Not detected | [36]^e^ |
|  | North Jetty | 26°55'06"S | 15°11'30"E | Not detected | [36]^e^ |
|  | Elizabeth Bay South | 26°56'08"S | 15°13'48"E | Not detected | [36]^e^ |
|  | Bogenfels | 27°27'26"S | 15°23'27"E | Not detected | [36]^e^ |
|  | Cabin | 27°36'26"S | 15°29'48"E | Not detected | [36]^e^ |
|  | Dernberg Bay | 27°42'37"S | 15°31'52"E | Not detected | [36]^e^ |
|  | Site 1 | 27°51'19"S | 15°38'52"E | Not detected | [36]^e^ |
|  | Panther Reef | 27°54'55"S | 15°41'05"E | Not detected | [36]^e^ |
|  | Hostel (H155-160) | 28°02'50"S | 15°45'56"E | Not detected | [52] |
|  | No. 1 Plant (H35-50) | 28°07'38"S | 15°51'00"E | Not detected | [52] |
|  | Kerbe Huk (K65-69) | 28°13'53"S | 15°59'01"E | Not detected | [52] |
|  | No. 2 Plant (M170-175) | 28°16'46"S | 16°02'45"E | Not detected | [52] |
|  | Mittag (M80-85) | 28°20'20"S | 16°06'24"E | Not detected | [52] |
|  | Oranjemund | 28°35'07"S | 16°23'28"E | Not detected | [6] |
|  |  |  |  |  |  |
| 2013 | Wolf Bay | 26°48'27"S | 15°07'27"E | Not detected | [36]^e^ |
|  | Atlas Bay | 26°49'45"S | 15°08'21"E | Not detected | [36]^e^ |
|  | West Whale | 26°54'32"S | 15°09'39"E | Not detected | [36]^e^ |
|  | East Whale | 26°54'28"S | 15°09'47"E | Not detected | [36]^e^ |
|  | Elizabeth Bay Point, exposed | 26°55'27"S | 15°11'31"E | Not detected | [36]^e^ |
|  | Elizabeth Bay Point, semi-exposed | 26°55'21"S | 15°11'32"E | Not detected | [36]^e^ |
|  | South Jetty | 26°55'13"S | 15°11'31"E | Not detected | [36]^e^ |
|  | North Jetty | 26°55'06"S | 15°11'30"E | Not detected | [36]^e^ |
|  | Elizabeth Bay South | 26°56'08"S | 15°13'48"E | Not detected | [36]^e^ |
|  | Bogenfels | 27°27'26"S | 15°23'27"E | Not detected | [36]^e^ |
|  | Cabin | 27°36'26"S | 15°29'48"E | Not detected | [36]^e^ |
|  | Dernberg Bay | 27°42'37"S | 15°31'52"E | Not detected | [36]^e^ |
|  | Site 1 | 27°51'19"S | 15°38'52"E | Not detected | [36]^e^ |
|  | Panther Reef | 27°54'55"S | 15°41'05"E | Not detected | [36]^e^ |
|  | Hostel (H155-160) | 28°02'50"S | 15°45'56"E | Not detected | [52] |
|  | No. 1 Plant (H35-50) | 28°07'38"S | 15°51'00"E | Not detected | [52] |
|  | Kerbe Huk (K65-69) | 28°13'53"S | 15°59'01"E | Not detected | [52] |
|  | No. 2 Plant (M170-175) | 28°16'46"S | 16°02'45"E | Not detected | [52] |
|  | Mittag (M80-85) | 28°20'20"S | 16°06'24"E | Not detected | [52] |
|  |  |  |  |  |  |
| 2014 | Wolf Bay | 26°48'27"S | 15°07'27"E | Not detected | [36]^e^ |
|  | Atlas Bay | 26°49'45"S | 15°08'21"E | Not detected | [36]^e^ |
|  | West Whale | 26°54'32"S | 15°09'39"E | Not detected | [36]^e^ |
|  | East Whale | 26°54'28"S | 15°09'47"E | Not detected | [36]^e^ |
|  | Elizabeth Bay Point, exposed | 26°55'27"S | 15°11'31"E | Not detected | [36]^e^ |
|  | Elizabeth Bay Point, semi-exposed | 26°55'21"S | 15°11'32"E | Not detected | [36]^e^ |
|  | South Jetty | 26°55'13"S | 15°11'31"E | Not detected | [36]^e^ |
|  | North Jetty | 26°55'06"S | 15°11'30"E | Not detected | [36]^e^ |
|  | Elizabeth Bay South | 26°56'08"S | 15°13'48"E | Not detected | [36]^e^ |
|  | Hostel (H155-160) | 28°02'50"S | 15°45'56"E | Not detected | [53] |
|  | No. 1 Plant (H35-50) | 28°07'38"S | 15°51'00"E | Not detected | [53] |
|  | Kerbe Huk (K65-69) | 28°13'53"S | 15°59'01"E | Not detected | [53] |
|  | No. 2 Plant (M170-175) | 28°16'46"S | 16°02'45"E | Not detected | [53] |
|  | Mining Licence Area 1 (M170-175) | 28°16'46"S | 16°02'45"E | Present | [6] |
|  | Mittag (M80-85) | 28°20'20"S | 16°06'24"E | Present | [53] |
|  |  |  |  |  |  |
| 2015 | Luanda | 8°48'S | 13°14'E | Present | [37]^a^ |
|  | Wolf Bay | 26°48'27"S | 15°07'27"E | Not detected | [36]^e^ |
|  | Atlas Bay | 26°49'45"S | 15°08'21"E | Not detected | [36]^e^ |
|  | West Whale | 26°54'32"S | 15°09'39"E | Not detected | [36]^e^ |
|  | East Whale | 26°54'28"S | 15°09'47"E | Not detected | [36]^e^ |
|  | Elizabeth Bay Point, exposed | 26°55'27"S | 15°11'31"E | Not detected | [36]^e^ |
|  | Elizabeth Bay Point, semi-exposed | 26°55'21"S | 15°11'32"E | Not detected | [36]^e^ |
|  | South Jetty | 26°55'13"S | 15°11'31"E | Not detected | [36]^e^ |
|  | North Jetty | 26°55'06"S | 15°11'30"E | Not detected | [36]^e^ |
|  | Elizabeth Bay South | 26°56'08"S | 15°13'48"E | Not detected | [36]^e^ |
|  | Hostel (H155-160) | 28°02'50"S | 15°45'56"E | Not detected | [54] |
|  | No. 1 Plant (H35-50) | 28°07'38"S | 15°51'00"E | Present | [54] |
|  | Kerbe Huk (K65-69) | 28°13'53"S | 15°59'01"E | Not detected | [54] |
|  | No. 2 Plant (M170-175) | 28°16'46"S | 16°02'45"E | Present | [54] |
|  | Mittag (M80-85) | 28°20'20"S | 16°06'24"E | Present | [54] |
|  |  |  |  |  |  |
| 2016 | Terrace Bay | 19°59'53"S | 13°01'58"E | Present | [6]^a^, [17]^a^ |
|  | Cape Cross | 21°45'23"S | 13°57'55"E | Present | [6]^a^, [17]^a^ |
|  | Langstrand | 22°48'32"S | 14°32'35"E | Present | [6]^a^, [17]^a^ |
|  | Pelican Point Jetty, Langstrand | 22°48'41"S | 14°32'33"E | Present | [6]^a^ |
|  | Wolf Bay | 26°48'27"S | 15°07'27"E | Not detected | [36]^e^ |
|  | Atlas Bay | 26°49'45"S | 15°08'21"E | Not detected | [36]^e^ |
|  | West Whale | 26°54'32"S | 15°09'39"E | Not detected | [36]^e^ |
|  | East Whale | 26°54'28"S | 15°09'47"E | Not detected | [36]^e^ |
|  | Elizabeth Bay Point, exposed | 26°55'27"S | 15°11'31"E | Not detected | [36]^e^ |
|  | Elizabeth Bay Point, semi-exposed | 26°55'21"S | 15°11'32"E | Not detected | [36]^e^ |
|  | South Jetty | 26°55'13"S | 15°11'31"E | Not detected | [36]^e^ |
|  | North Jetty | 26°55'06"S | 15°11'30"E | Not detected | [36]^e^ |
|  | Elizabeth Bay South | 26°56'08"S | 15°13'48"E | Not detected | [36]^e^ |
|  | Hostel (H155-160) | 28°02'50"S | 15°45'56"E | Not detected | [55] |
|  | No. 1 Plant (H35-50) | 28°07'38"S | 15°51'00"E | Not detected | [55] |
|  | Kerbe Huk (K65-69) | 28°13'53"S | 15°59'01"E | Present | [55] |
|  | No. 2 Plant (M170-175) | 28°16'46"S | 16°02'45"E | Present | [55] |
|  | Mittag (M80-85) | 28°20'20"S | 16°06'24"E | Not detected | [55] |
|  |  |  |  |  |  |
| 2017 | Langstrand | 22°49'24"S | 14°32'33"E | Present | [67] |
|  | Wolf Bay | 26°48'27"S | 15°07'27"E | Not detected | [36]^e^ |
|  | Atlas Bay | 26°49'45"S | 15°08'21"E | Not detected | [36]^e^ |
|  | West Whale | 26°54'32"S | 15°09'39"E | Not detected | [36]^e^ |
|  | East Whale | 26°54'28"S | 15°09'47"E | Not detected | [36]^e^ |
|  | Elizabeth Bay Point, exposed | 26°55'27"S | 15°11'31"E | Not detected | [36]^e^ |
|  | Elizabeth Bay Point, semi-exposed | 26°55'21"S | 15°11'32"E | Not detected | [36]^e^ |
|  | South New Jetty | 26°55'16"S | 15°11'34"E | Not detected | [36]^e^ |
|  | South Jetty | 26°55'13"S | 15°11'31"E | Not detected | [36]^e^ |
|  | North Jetty | 26°55'06"S | 15°11'30"E | Present | [36]^e^ |
|  | Elizabeth Bay South | 26°56'08"S | 15°13'48"E | Not detected | [36]^e^ |
|  | Hostel (H155-160) | 28°02'50"S | 15°45'56"E | Present | [56] |
|  | No. 1 Plant (H35-50) | 28°07'38"S | 15°51'00"E | Not detected | [56] |
|  | Kerbe Huk (K65-69) | 28°13'53"S | 15°59'01"E | Present | [56] |
|  | No. 2 Plant (M170-175) | 28°16'46"S | 16°02'45"E | Present | [56] |
|  | Mittag (M80-85) | 28°20'20"S | 16°06'24"E | Present | [56] |
|  |  |  |  |  |  |
| 2018 | Wolf Bay | 26°48'27"S | 15°07'27"E | Not detected | [36]^e^ |
|  | Atlas Bay | 26°49'45"S | 15°08'21"E | Not detected | [36]^e^ |
|  | West Whale | 26°54'32"S | 15°09'39"E | Present | [36]^e^ |
|  | East Whale | 26°54'28"S | 15°09'47"E | Not detected | [36]^e^ |
|  | Elizabeth Bay Point, exposed | 26°55'27"S | 15°11'31"E | Not detected | [36]^e^ |
|  | Elizabeth Bay Point, semi-exposed | 26°55'21"S | 15°11'32"E | Present | [36]^e^ |
|  | South New Jetty | 26°55'16"S | 15°11'34"E | Present | [36]^e^ |
|  | South Jetty | 26°55'13"S | 15°11'31"E | Present | [36]^e^ |
|  | North Jetty | 26°55'06"S | 15°11'30"E | Not detected | [36]^e^ |
|  | Elizabeth Bay South | 26°56'08"S | 15°13'48"E | Not detected | [36]^e^ |
|  | Hostel (H155-160) | 28°02'50"S | 15°45'56"E | Present | [57] |
|  | No. 1 Plant (H35-50) | 28°07'38"S | 15°51'00"E | Present | [57] |
|  | Kerbe Huk (K65-69) | 28°13'53"S | 15°59'01"E | Present | [57] |
|  | No. 2 Plant (M170-175) | 28°16'46"S | 16°02'45"E | Present | [57] |
|  | Mittag (M80-85) | 28°20'20"S | 16°06'24"E | Present | [57] |
|  |  |  |  |  |  |
| 2019 | Portuguese Lorry North | 17°33'53"S | 11°44'12"E | Not detected | [20] |
|  | Portuguese Lorry South | 17°35'08"S | 11°44'11"E | Not detected | [20] |
|  | Angra Fria | 18°17'09"S | 11°57'47"E | Not detected | [20] |
|  | False Cape Fria North | 18°28'51"S | 12°01'26"E | Present | [20] |
|  | False Cape Fria South | 18°29'04"S | 12°01'34"E | Present | [20] |
|  | Rocky Point, sheltered | 18°59'39"S | 12°28'35"E | Not detected | [20] |
|  | Rocky Point Bench 1, exposed | 18°59'41"S | 12°28'31"E | Present | [20] |
|  | Rocky Point Bench 2, exposed | 18°59'41"S | 12°28'32"E | Present | [20] |
|  | North of Möwe Bay, sheltered | 19°20'54"S | 12°42'24"E | Not detected | [20] |
|  | Möwe Bay Bench 1, semi-exposed | 19°22'54"S | 12°42'16"E | Present | [20] |
|  | Möwe Bay Bench 2, exposed | 19°22'54"S | 12°42'16"E | Present | [20] |
|  | Student Bay Bench 1 | 20°08'01"S | 13°07'33"E | Present | [20] |
|  | Student Bay Bench 2 | 20°08'01"S | 13°07'33"E | Present | [20] |
|  | South of Möwe Bay, seal colony | 20°08'02"S | 13°07'04"E | Present | [20] |
|  | Henties Bay, Solitude South | 22°09'03"S | 14°17'17"E | Present | [21] |
|  | Mile Four | 22°37'08"S | 14°31'15"E | Not detected | [21] |
|  | Patrysberg | 22°44'28"S | 14°31'28"E | Present | [21] |
|  | Langstrand, Swakopmund | 22°49'02"S | 14°32'31"E | Present | [21] |
|  | Meob | 24°32'12"S | 14°35'55"E | Present | [22] |
|  | Spencer Bay North | 25°37'19"S | 14°50'55"E | Present | [22] |
|  | Spencer Bay South | 25°44'04"S | 14°50'52"E | Present | [22] |
|  | Diaz Point, sheltered | 26°38'36"S | 15°05'26"E | Not detected | [22] |
|  | Diaz Point, semi-exposed | 26°39'17"S | 15°04'57"E | Not detected | [22] |
|  | Grossebucht, semi-exposed | 26°44'23"S | 15°05'26"E | Not detected | [22] |
|  | Grossebucht, sheltered | 26°44'18"S | 15°05'40"E | Not detected | [22] |
|  | Wolf Bay | 26°48'27"S | 15°07'27"E | Not detected | [36]^e^ |
|  | Atlas Bay | 26°49'45"S | 15°08'21"E | Not detected | [36]^e^ |
|  | West Whale | 26°54'32"S | 15°09'39"E | Present | [36]^e^ |
|  | East Whale | 26°54'28"S | 15°09'47"E | Not detected | [36]^e^ |
|  | Elizabeth Bay Point, exposed | 26°55'27"S | 15°11'31"E | Not detected | [36]^e^ |
|  | Elizabeth Bay Point, semi-exposed | 26°55'21"S | 15°11'32"E | Present | [36]^e^ |
|  | South New Jetty | 26°55'16"S | 15°11'34"E | Not detected | [36]^e^ |
|  | South Jetty | 26°55'13"S | 15°11'31"E | Not detected | [36]^e^ |
|  | North Jetty | 26°55'06"S | 15°11'30"E | Not detected | [36]^e^ |
|  | Elizabeth Bay South | 26°56'08"S | 15°13'48"E | Not detected | [36]^e^ |
|  |  |  |  |  |  |

^a^ Date of publication

^b^ Dates were inferred from the authors’ description of their visits to rocks 5 km south of the Kunene River mouth

^c^ Date of collection provided by G.M. Branch

^d^ Survey was done by the Namibian Ministry of Fisheries and Marine Resources

^e^ Data originally from the Namdeb Diamond Corporation’s long-term coastal biological monitoring programme
